# Chromium Affects Mitochondrial Function, Leading to Apoptosis and Autophagy in Turtle Primary Hepatocytes

**DOI:** 10.3390/ani14162403

**Published:** 2024-08-19

**Authors:** Shuqin Lin, Yunjuan Xiao, Jing Lin, Yue Yuan, Haitao Shi, Meiling Hong, Li Ding

**Affiliations:** Ministry of Education Key Laboratory for Ecology of Tropical Islands, Key Laboratory of Tropical Animal and Plant Ecology of Hainan Province, College of Life Sciences, Hainan Normal University, Haikou 571158, China

**Keywords:** hexavalent chromium, primary hepatocytes, oxidative stress, mitochondrial dysfunction, cell death

## Abstract

**Simple Summary:**

Hexavalent chromium (Cr(VI)) poses a considerable threat to aquatic ecosystems and aquatic organisms due to pollution. This study explored the toxic effects of Cr(VI) on the primary hepatocytes of the Reeves’ turtles (*Mauremys reevesii*) by isolating the hepatocytes. The results showed that Cr(VI)-treated cells exhibited increased antioxidant enzyme activity and reactive oxygen species levels. ATP levels decreased, and related mitochondrial dynamics genes underwent changes. Ultimately, this led to apoptosis and autophagy in the cells. These findings indicate that Cr(VI) pollution is a critical health issue, offering fresh perspectives and guiding principles for the protection of turtles.

**Abstract:**

Hexavalent chromium (Cr(VI)), a pervasive industrial contaminant, is highly toxic to both humans and animals. However, its effects on turtles are largely unexplored. Our study aimed to investigate the toxic effects of Cr(VI) on the Reeves’ turtles (*Mauremys reevesii*) primary hepatocytes. We exposed hepatocytes to two concentrations (25 μM and 50 μM) of Cr(VI) for 24 h. The results showed that compared to controls, Cr(VI)-treated cells showed elevated antioxidant enzyme activity (catalase (CAT) and superoxide dismutase (SOD)) and increased reactive oxygen species (ROS) levels. Adenosine triphosphatae (ATP) levels decreased, indicating mitochondrial dysfunction. Additionally, we found significant changes in mitochondrial dynamics related genes, with downregulation of mitofusin 2 (Mfn2) and silent information regulator 1 (SIRT1) and a decrease in sirtuin 3 (SIRT3) and tumor protein 53 (p53) mRNA levels. Annexin V-FITC fluorescence staining-positive cells increased with higher Cr(VI) concentrations, marked by elevated bcl-2-associated X protein (Bax) and cysteinyl aspartate specific proteinase (Caspase3) mRNA levels and reduced B-cell lymphoma-2 (Bcl2) expression. Autophagy-related genes were also affected, with increased microtubule-associated protein 1 light chain 3 (LC3-I), microtubule-associated protein light chain 3II (LC3-II), unc-51-like autophagy-activating kinase 1 (ULK1), and sequestosome 1 (p62/SQSTM1) mRNA levels and decreased mammalian target of rapamycin (mTOR) and Beclin1 expression. Taken together, Cr(VI) promotes cell apoptosis and autophagy in turtle hepatocytes by inducing oxidative stress and disrupting mitochondrial function. These findings highlight the serious health risks posed by Cr(VI) pollution and emphasize the need for protecting wild turtle populations.

## 1. Introduction

Hexavalent chromium (Cr(VI)) denotes chromium metal present in the form of 6+ ions. This particular form is highly toxic and poses significant health hazards. Considerable quantities of hexavalent chromium are generated during various industrial processes, including electroplating, stainless steel production, leather tanning, textile manufacturing, and the creation of plastics, fabric coloring, coatings, and inks. Industrial waste from coal-fired power stations also contributes to the production of this harmful substance [1,2,3]. Cr(VI), with its potent properties, poses significant challenges in the environment, as it is notoriously difficult to eliminate. Its biomagnification through the food chain and bioaccumulation via oral ingestion, inhalation, and prolonged skin contact pose a substantial threat to human health. Prolonged exposure can lead to severe damage to the respiratory tract, digestive system, and other vital organs [4]. Characterized by high solubility, strong mobility, toxicity, and corrosiveness, Cr(VI) exhibits stimulating, mutagenic, and carcinogenic effects once it permeates cells via transmembrane transport [5,6,7]. Due to its toxic effects, the International Center for Research on Cancer has classified Cr(VI) as a Class 1 carcinogen [8].

The harm caused by Cr(VI) primarily originates from the leakage and dispersal of hexavalent chromium in soil, posing significant pollution threats to the ecological environment [9]. The chief concern, however, lies in the pronounced alkalinity of chromium slag. When this slag is discarded in outdoor storage and exposed to rainfall, a substantial amount of hexavalent chromium ion infiltrates the soil along with the rainwater. This not only contaminates groundwater but also extends its reach to lakes, rivers, and irrigation systems for farmland. Consequently, contaminated water finds its way into agricultural produce and aquatic life, ultimately posing a risk to the health of both humans and animals [3,10,11]. Chemical wastewater often contains very high levels of hexavalent chromium. When released untreated, it can easily seep into the groundwater, elevating hexavalent chromium levels dangerously. Reports indicate that in certain regions of the United States, such as Oregon, groundwater chromium concentration has reached alarming levels of up to 14,600 mg/L, with the soil chromium content soaring up to 25,900 mg/kg [12]. Similarly, in Hazaribagh, an industrial zone in Bangladesh’s capital Dhaka, soil chromium levels have been recorded at 150 mg/kg, surpassing the European Union’s maximum permissible limit. Moreover, the chromium concentration in deep groundwater has also exceeded the strictest limit set by the national department of energy (10 μg/L) [13]. In China, the recently implemented “Sanitary Standard for Drinking Water” (GB5749-2022) [14] in 2023 has set a strict limit of Cr(VI) in drinking water, reflecting the gravity of the situation and the need for stringent measures. The pollution caused by chromium-containing industrial wastewater has resulted in significant excesses of Cr(VI) in groundwater and drinking water in numerous cities across the country. According to Peng et al. [15], the concentration of Cr in Xiangjiang River has surpassed the acceptable limits for urban drinking water. The accumulation of chromium waste residue, coupled with the continuous discharge of chromium-containing wastewater, continues to pollute the soil and groundwater in the surrounding environment. This persistent pollution has led to elevated chromium concentrations that exceed safety standards, posing a substantial threat to the health and well-being of nearby humans, animals, and plants [16].

Cr(VI) enters the body primarily through the digestive tract, respiratory tract, and skin mucous membrane. Once inside, it accumulates in organs such as the liver, kidneys, and endocrine glands, resulting in chronic damage to these and other target organs [4]. Cr(VI) penetrates cells via cell membrane ion channels and gets gradually reduced into Cr(V), Cr(IV), and Cr(III) within the cell [17]. Cr(III) is not easily expelled from the cell and can accumulate over the long term, interacting with biological macromolecules and causing continuous cellular damage [18,19]. In this process, various forms of reactive oxygen species (ROS), including superoxide and hydrogen peroxide, are produced [20]. The excessive generation of ROS can lead to oxidative stress and DNA damage, ultimately resulting in cell apoptosis and necrosis [21,22]. Animal experiments have shown that exposure to hexavalent chromium can induce oxidative stress and tissue damage in the mitochondria and microsomes of rat liver and brain cells [23]. The liver, as a crucial site for metabolism and detoxification in the body, is a primary target organ for Cr(VI) toxicity [22]. Previous research has indicated that long-term exposure to Cr(VI) can affect the antioxidant system, triggering oxidative stress and DNA damage by activating or disrupting this system [8]. In a study where goldfish (*Carassius auratus*) were exposed to varying doses of Cr(VI) for 96 h, observations revealed loosely arranged and vacuolated liver cells, the appearance of giant nuclei, distorted and damaged central veins, as well as atrophy and degeneration of hepatocytes along with central vein injury central vein injury [24].

As an integral component of the aquatic ecosystem, the Reeves’ turtles (*Mauremys reevesii*) serves as a valuable indicator water environmental pollution. For turtles, chromium exposure primarily occurs through the ingestion of contaminated food or direct skin contact with polluted water. Chromium exhibits a tendency to accumulate and concentrate within the biological chain. Once passively absorbed by plants, chromium migrates and accumulates in various organisms [4]. Currently, research on the effects of Cr(VI) on turtles remains limited. Previous studies have shown that the cytotoxic and genotoxic effects of Cr(VI) on marine turtles, specifically on lung fibroblasts of leatherback sea turtle (*Dermochelys coriacea*) and skin fibroblasts of hawksbill sea turtle (*Eretmochelys imbricata*) [25,26]. However, such findings have not been documented for freshwater turtles. Hence, this study utilizes primary isolated hepatocytes from the freshwater-dwelling vertebrate Reeves’ turtles (*Mauremys reevesii*) to investigate the impact of Cr(VI) exposure on hepatocyte toxicity, including oxidative stress, mitochondrial dynamic balance, apoptosis, and autophagy.

## 2. Materials and Methods

### 2.1. Chemicals and Reagents

Potassium chromate (K_2_CrO_4_) was purchased from Shanghai Macklin Biochemical Technology Co., Ltd. (Shanghai, China). All primers were expertly synthesized by Sangon Biotech Co., Ltd. (Shanghai, China). Annexin V-FITC/PI double-dyed apoptosis detection kits, adenosine triphosphatae (ATP) content test kits, catalase (CAT), and superoxide dismutase (SOD) kits were purchased from Nanjing Jiancheng Bioengineering Institute (Nanjing, China). Reactive oxygen species (ROS) kits were procured from Wuhan Servicebio Technology Co., Ltd. (Wuhan, China). For cell cultivation, we utilized high-glucose DMEM media provided by Thermo Fisher Scientific (Waltham, MA, USA). Additionally, we obtained Australian fetal bovine serum through Cell-Box (HK) Biological products Trading Co., Ltd. (Changsha, China).

### 2.2. Cell Culture and Processing

Liver tissue collection was conducted as follows: A dying Reeves’ turtle was found in the wild but succumbed to its illness despite attempts at treatment. Immediately upon its demise, the turtle underwent disinfection and dissection procedures. The liver was carefully extracted and washed thoroughly three times in PBS containing 4% triple antibiotic (penicillin-streptomycin-amphotericin B) to eliminate as much blood and clots as possible. The liver was then placed in a fresh cell culture dish, and using precision eye scissors, the tissue was finely minced. The minced tissue was transferred to a 15 mL centrifuge tube and was centrifuged at 500 rpm for 5 min, discarding the supernatant. An appropriate amount of trypsin was added for digestion, and the mixture was placed in a 37 °C water bath for 30 min. Once digestion was complete, DMEM with 10% serum was added to halt the digestion process. The mixture was then poured through a 70 μM filter membrane. The filtered liquid was collected in 15 mL centrifuge tubes and centrifuged at 1000 rpm for 5 min. After discarding the supernatant, DMEM medium containing penicillin streptomycin (1×), amphotericin B (1×), and 10% FBS was added to suspend the cells. These were then carefully transferred to T25 cell culture flasks and incubated in a controlled environment at 30 °C, 5% CO_2_ [27,28,29]. The apparatus used in the present experiment is sterile. The experiment was approved by the Animal Research Ethics Committee of Hainan Provincial Education Center for Ecology and Environment, Hainan Normal University (HNECEE-2023-005).

### 2.3. Cell Growth Assay

Cells were digested into individual cells, enumerated using a counting plate, and seeded into 96-well plates at a density of 5000 cells/well. The cultures were set up using media containing 10% and 15% serum, respectively. The incubation time was 1 to 7 days, with cellular activity assessed every 24 h via the Cell Counting Kit-8 (CCK8) provided by Beyotime (Shanghai, China). Specifically, 10 μL of CCK-8 solution was added to each culture well and incubated at 37 °C for 2 h. The absorbance was measured at 450 nm using an enzyme marker.

### 2.4. Cytotoxicity Assay

Cells were seeded in 96-well plates at a density of 10,000 cells per well. These cells were then cultured in media containing varying concentrations of hexavalent chromium Cr(VI): 0 μM, 10 μM, 20 μM, 30 μM, 40 μM, 50 μM, and 60 μM, respectively. Each concentration was repeated 3 times. The cells were exposed to this stress environment for a full 24 h. To assess cytotoxicity, CCK8 assay was employed. Specifically, 10 μL of CCK-8 solution was added to each well, and the plates were incubated at 37 °C for 2 h. Following incubation, the absorbance was measured at 450 nm using an enzyme marker.

### 2.5. Oxidative Stress Detection

Cells were cultured until they reached over 80% in 6-well plates. The medium was then replaced with cultures containing 0 μM, 25 μM, and 50 μM hexavalent chromium Cr(VI), respectively. After 24 h of culture, the cells were gently scraped with a cell scraper and collected into EP tubes. Homogenate protein concentration was measured using the Bradford assay protein quantitative detection kit of Wuhan Servicebio Technology Co., Ltd. (Wuhan, China). Catalase (CAT) decomposed H_2_O_2_ and acted with ammonium molybdate to produce a light-yellow complex. The optical absorption of this complex was measured at 405 nm. Additionally, the WST-1 method was used to measure superoxide dismutase (SOD) activity. In this assay, WST-1 reacted with xanthine oxidase to produce mazan dye, which was inhibited by the dismutation of superoxide anion catalyzed by SOD. The optical absorption value was determined at 450 nm.

### 2.6. Reactive Oxygen Species (ROS)

The ROS detection kit utilizes the fluorescent probe DCFH-DA for reactive oxygen species. Initially non-fluorescent, DCFH-DA effortlessly permeates the cell membrane, where it is hydrolyzed by esterase to form DCFH. Inside the cell, DCFH can be oxidized by ROS to yield fluorescent DCF. Importantly, the fluorescence signal strength directly correlates with the intracellular ROS level. In order to conduct the experiment, healthy cells were seeded into a 6-well plate a day prior, ensuring proper adherence. These cells were then exposed to 0 μM, 25 μM, and 50 μM Cr(VI) medium for 24 h. Following incubation, the medium was discarded, and the cells were rinsed twice with PBS. DCFH-DA working solution was introduced, and the cells were incubated in a CO_2_ incubator at 37 °C for 30 min in darkness. The DCFH-DA solution was removed, the cells were washed three times with PBS, and they were finally imaged under a fluorescence microscope.

### 2.7. Apoptosis Detection

Phosphatidylserine (PS) is primarily located on the inner leaflet of the cell membrane. However, during the early stage of apoptosis, cells relocate phosphatidylserine to the cell surface. Annexin V exhibits a strong affinity for phosphatidylserine exposed on the cell surface. Annexin V-FITC apoptosis kits utilize FITC-labeled recombinant Annexin V for the detection of phosphatidylserine present on the cell membrane surface during apoptosis. To conduct the experiment, a coverslip was positioned in a 6-well plate, and the cells were cultivated on this coverslip in media containing 0 μM, 25 μM, and 50 μM hexavalent chromium Cr(VI) for 24 h. After incubation, the culture medium was aspirated, and the cells were rinsed twice with PBS. Annexin V-FITC solution was then applied to the culture wells and incubated in the dark at room temperature for 10 min. The cells were subsequently observed and photographed using a fluorescence microscope.

### 2.8. ATP Level Detection

Adenosine triphosphate (ATP) serves as the fundamental medium for energy conversion within the body. Typically, when a cell undergoes apoptosis, necrosis, or experiences a toxic state, its ATP level diminishes. This reduction in ATP signifies potential damage or impairment to mitochondrial function. To assess this, cell suspensions were subjected to a boiling water bath for 10 min, followed by a mixing period of 1 min, and were subsequently evaluated as per the provided instructions.

### 2.9. qRT-PCR Analysis

After incubating cells in 6-well plates for 24 h, the medium was discarded, and the cells were washed twice with PBS; 1 mL TRIzol was added to each well, mixed, and lysed for 10 min at room temperature. The cell lysate was transferred to a 1.5 mL EP tube, mixed with 0.2 mL chloroform, incubated for 10 min, and centrifuged at 12,000 r/min at 4 °C for 15 min. The supernatant was transferred to a new DEPC-treated EP tube, mixed with equal volumes of isopropyl alcohol, incubated for 10 min, and centrifuged again. The supernatant was discarded, and the RNA precipitate was washed with 75% ethanol in DEPC water and centrifuged. After discarding the supernatant, the precipitate was dried and dissolved in 30 μL DEPC water. RNA integrity and concentration were detected by agarose gel electrophoresis and nanodrop, respectively. The specific primers (β-actin, B-cell lymphoma-2 (Bcl-2), bcl-2-associated X protein (Bax), cysteinyl aspartate-specific proteinase (Caspase3), sirtuin 3 (SIRT3), silent information regulator 1 (SIRT1), mammalian target of rapamycin (mTOR), mitofusin 2 (Mfn2), tumor protein 53 (p53), microtubule-associated proteins light chain 3II (LC3-II), microtubule-associated protein 1 light chain 3 (LC3-I), unc-51-like autophagy-activating kinase 1 (ULK1), sequestosome 1 (p62/SQSTM1), and Beclin-1) (Supplementary Material Table S1) were designed via NCBI-BLAST and synthesized by Sangon Biotech Co., Ltd. cDNA was reverse-transcribed using a kit from Tiangen Biochemical Technology Co., Ltd. (Beijing, China), and gene expression was quantified using a Roche Light Cycler 480II (Basel, Switzerland). The relative expression profile of selected genes was calculated by the 2^−ΔΔct^.

### 2.10. Statistic Analysis

Data are expressed as mean ± SEM. After testing normality and homogeneity with SPSS20.0 software, the differences between groups were analyzed by one-way analysis of variance (ANOVA) and the LSD multiple comparison method. The significance level was set at *p* < 0.05.

## 3. Results

### 3.1. Cell Growth Curve and Morphology of Primary Hepatocytes

**Figure 1:**
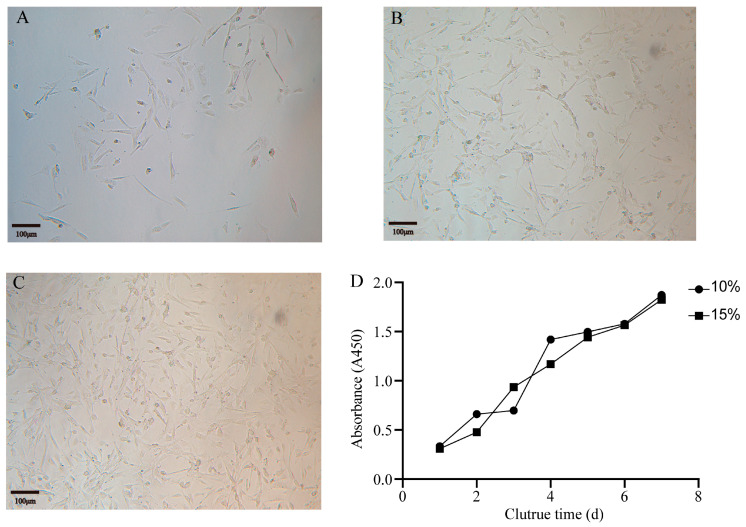
The primary cell culture and growth curve. (**A**) After 3 days of culture, it showed a small amount of adherent cell growth. (**B**) Cells on day 2 after the first passage. (**C**) Cell morphology after the fifth passage. (**D**) Cell viability.

After digestion, the turtle hepatocytes were inoculated into cell culture vials. Following a 72 h incubation, a small number of cells had adhered to the vial wall (Figure 1A). Most of these cells exhibited a spindle-shaped and irregular morphology, characterized by a uniform cytoplasm with minimal impurities. During the first to fifth passages, the cells underwent rapid proliferation while maintaining morphological uniformity (Figure 1B,C). To further investigate the growth kinetics, the cells were seeded into 96-well plates at a density of 5000 cells per well. Cell growth curves were plotted using the CCK-8 assay, allowing for the comparison of different serum concentrations. The hepatocytes were cultured for 1 to 7 days in media containing either 10% or 15% serum (Figure 1D). Notably, all turtle hepatocytes were able to enter an exponential growth phase, with their growth rate progressively increasing over time. However, the variation in serum concentration had minimal impact on their growth patterns. Consequently, in subsequent experiments, a 10% serum concentration was chosen for cell culture.

### 3.2. Cr(VI) Inhibited the Growth of Turtle Hepatocytes

**Figure 2:**
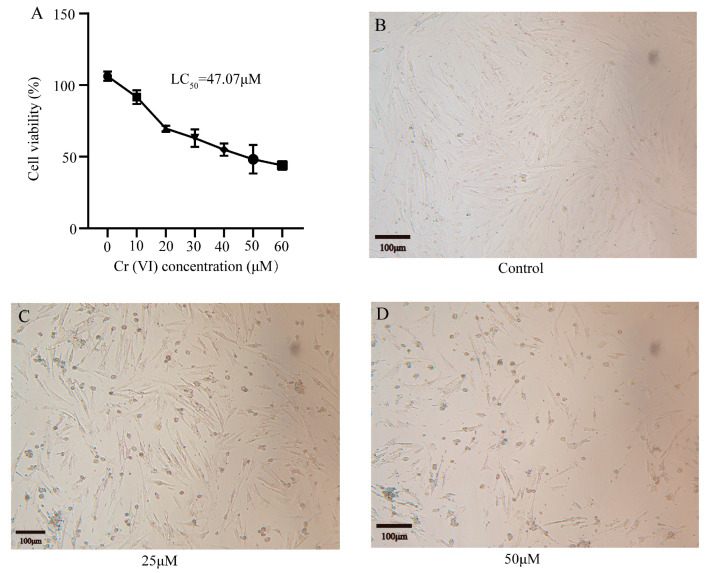
The effects of Cr(VI) on the cytotoxicity and morphology of hepatocytes. (**A**) Cytotoxic effect of Cr(VI) on *Mauremys reevesii* liver cells treated for 24 h. Data expressed as the percentage of cell viability. (**B**–**D**) The morphological changes of turtle hepatocytes treated with different concentrations of Cr(VI) for 24 h were observed under a microscope (scale: 100 μm).

The cells were cultivated in media containing various concentrations of Cr(VI), specifically 0 μM, 10 μM, 20 μM, 30 μM, 40 μM, 50 μM, and 60 μM. After 24 h of treatment, Cr(VI) exhibited pronounced cytotoxicity to turtle hepatocytes in a concentration-dependent manner (Figure 2A). The median lethal concentration (LC_50_) of Cr(VI) in hepatocytes was determined to be 47.07 μM. In order to further study the harmful effects of Cr(VI) on hepatocytes, we handpicked concentrations of 0 μM (serving as the control), 25 μM, and 50 μM for subsequent experiments. Cells were incubated in these selected concentrations of Cr(VI) for 24 h, and the morphology was observed under an inverted microscope. Compared with the control group, the number of adherent cells declined in both the 25 μM and 50 μM groups, correlating with the increase in Cr(VI) concentration. In addition, cells in the 25 μM and 50 μM groups exhibited a rounder shape, appeared to have shrunk, lost their ability to adhere to the cell wall, displayed irregular edges, and had enlarged intercellular spaces (Figure 2B–D).

### 3.3. Cr(VI) Induced Oxidative Stress in Turtle Hepatocytes

**Figure 3:**
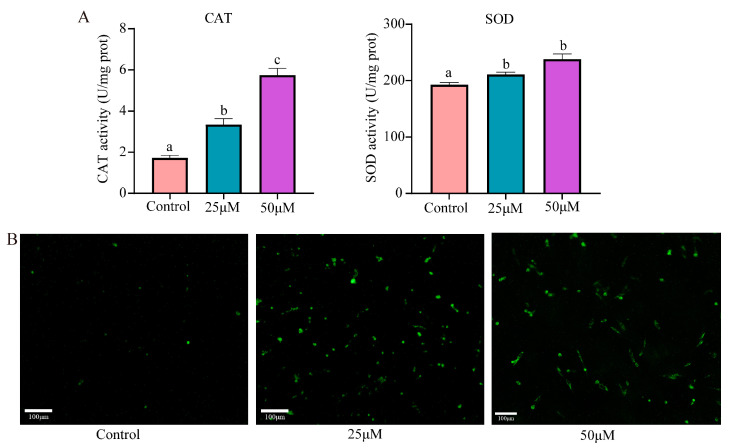
Effects of Cr(VI) on oxidative stress in turtle hepatocytes. (**A**) CAT and SOD activity levels. Data are expressed as the mean ± SEM. Values not sharing a common superscript letter differ significantly at *p* < 0.05. (**B**) Cellular ROS detection.

Following exposure to Cr(VI), the activity levels of CAT and SOD were increased in the 25 μM and 50 μM concentration groups when compared with the untreated control group. Specifically, CAT activity saw a notable surge, increasing significantly by 94.39% and 234.15% in the 25 μM and 50 μM groups, respectively. Similarly, SOD activity rose significantly by 9.41% and 23.58% in these groups (Figure 3A). The intracellular content of ROS also increased, revealing a dose-dependent change in green fluorescence (Figure 3B). This finding suggests that Cr(VI) stimulated an increase in the intracellular ROS level, indicating a heightened oxidative stress response within the cells.

### 3.4. Cr(VI) Decreased Mitochondrial Dynamics in Hepatocytes

**Figure 4:**
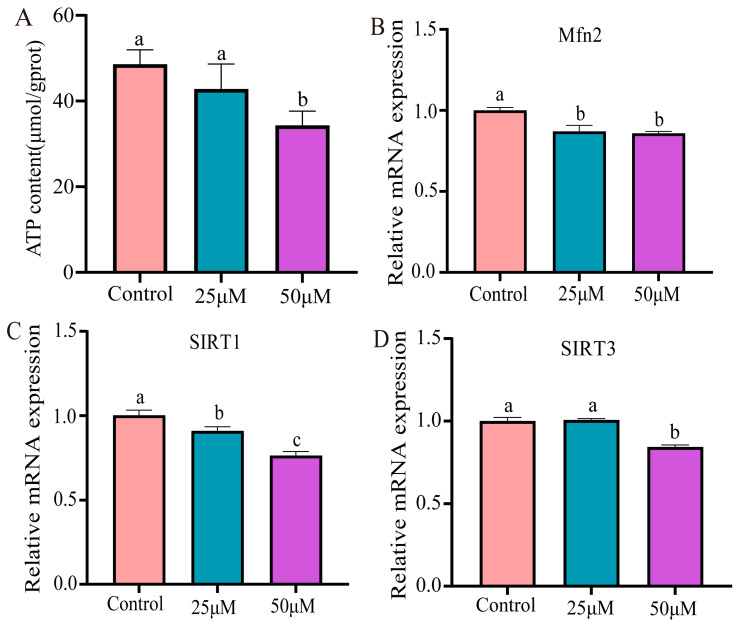
The mitochondrial dynamics in turtle hepatocyte. (**A**) ATP production levels. (**B**–**D**) Mitochondrial dynamics-related mRNA level (Mfn2, SIRT1, and SIRT3). Data are expressed as the mean ± SEM. Values not sharing a common superscript letter differ significantly at *p* < 0.05.

Through the detection of the mitochondrial status of hepatocytes exposed to Cr(VI), it was found that ATP production levels declined as the concentration of Cr(VI) increased. Notably, the 50 μM concentration group exhibited a significant decrease in mitochondrial ATP production (*p* < 0.05) (Figure 4A). Furthermore, mRNA levels were examined for genes involved in mitochondrial dynamics. Compared with the control group, the mRNA levels of Mfn2 and SIRT1 were significantly decreased in both the 25 μM and 50 μM group (*p* < 0.05) (Figure 4B,C). Additionally, the mRNA levels of SIRT3 showed a significant decrease, especially in the 50 μM group (Figure 4D).

### 3.5. Cr(VI) Activated the p53 Signal Pathway in the Liver Cells

**Figure 5:**
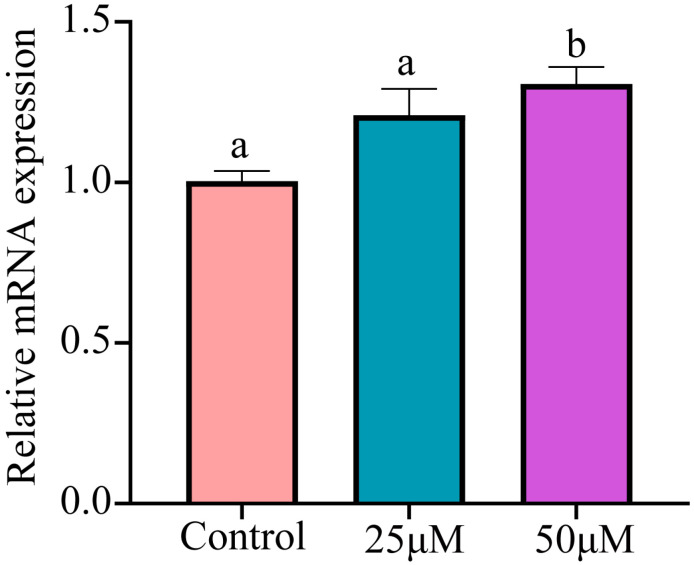
Cr(VI) activates the p53 pathway in turtle hepatocytes. Data are expressed as the mean ± SEM. Values not sharing a common superscript letter differ significantly at *p* < 0.05.

p53 can induce cell cycle arrest or apoptosis, and it regulates autophagy as a reaction to diverse cellular stress signals. Upon investigation, the mRNA level of the p53 gene was found to increase in a dose-dependent fashion, with a notable elevation observed at a concentration of 50 μM (*p* < 0.05) (Figure 5).

### 3.6. Cr(VI) Induced Apoptosis in Turtle Hepatocytes

**Figure 6:**
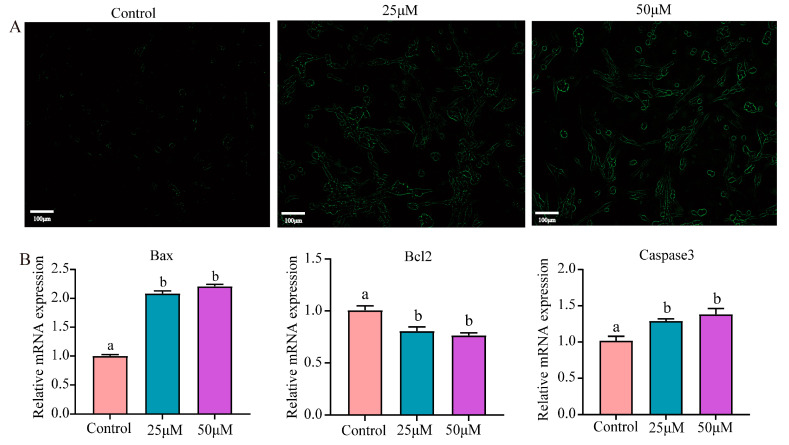
Effect of Cr(VI) on the apoptosis of turtle hepatocytes. (**A**) Fluorescence staining for apoptosis with Annexin V-FITC. (**B**) Apoptosis-related gene (Bax, Bcl2 and Caspase3) mRNA levels. Data are expressed as the mean ± SEM. Values not sharing a common superscript letter differ significantly at *p* < 0.05.

Apoptosis was detected by using the Annexin V-FITC probe, which emits green fluorescence, highlighting apoptotic cells as they stain green on the cell membrane surface. Evidently, as illustrated in Figure 6A, the treatment group exhibited stronger fluorescence, indicating a notable surge in apoptosis. In addition, in comparison to the control group, the mRNA levels of Bax and Caspase3 in the stress group significantly increased (*p* < 0.05), whereas the mRNA levels of Bcl2 experienced a considerable decline (*p* < 0.05) (Figure 6B).

### 3.7. Cr(VI) Caused Autophagy in Hepatocyte

**Figure 7:**
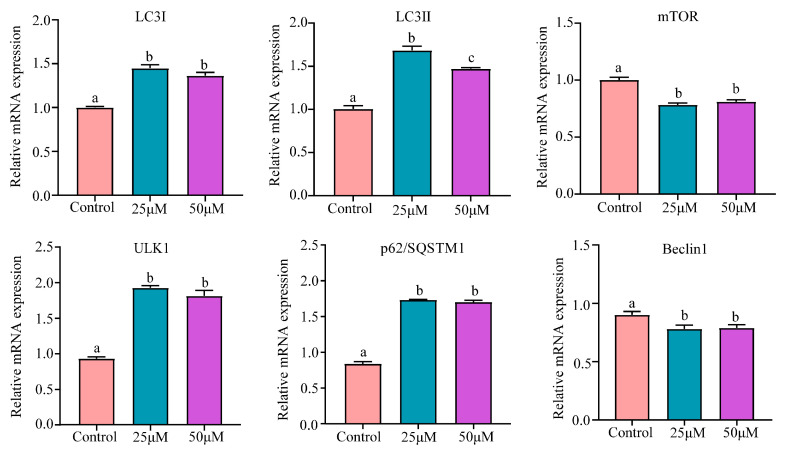
Effect of Cr(VI) on the autophagy of turtle hepatocytes. Data are expressed as the mean ± SEM. Values not sharing a common superscript letter differ significantly at *p* < 0.05.

To delve deeper into the effect of Cr(VI) on autophagy in hepatocytes, we conducted an assessment of autophagy-related mRNA expression levels. As clearly demonstrated in Figure 7, in comparison to the control group, the mRNA levels of autophagy-related genes LC3I and LC3II exhibited a significant surge in both the 25 μM and 50 μM groups (*p* < 0.05). Conversely, the mRNA levels of mTOR underwent a notable decline (*p* < 0.05). Additionally, there was a significant elevation in the mRNA levels of ULK1 and p62/SQSTM1 (*p* < 0.05), whereas the mRNA levels of Beclin-1 showed a considerable drop (*p* < 0.05).

## 4. Discussion

The escalating emission of gases and discharge of industrial wastewater containing anthropogenic Cr(VI) have significantly impacted wildlife health [30]. Turtles are an important part of wildlife; despite this, the toxic effects of Cr(VI) on turtles have garnered minimal attention. Consequently, we have conducted an investigation into the deleterious effects of Cr(VI) on turtle hepatocytes, utilizing primary hepatocytes from turtles as our experimental subjects. We found that Cr(VI) exhibited a very significant toxic effect on turtle primary hepatocytes.

Various studies have indicated that exposure to Cr(VI) stress results in the decreased activity of enzymes such as T-SOD, SOD, GSH-PX, GSH, and CAT in duck (*Anas platyrhyncha*) liver tissue. Simultaneously, it elevates the levels of MDA and H_2_O_2_, leading to oxidative stress [31]. Oxidative stress arises from an imbalance of free radicals in the body [32]. Catalase (CAT) and superoxide dismutase (SOD) are crucial antioxidant enzymes that form the backbone of the biological defense system, providing organisms with a vital antioxidant protection mechanism [33]. An excess of free radicals in the body often disrupts the normal functioning of antioxidant enzymes. Mitochondria are the primary site for the generation of reactive oxygen species (ROS) [34]. Under normal conditions, low concentrations of ROS in cells can exert beneficial effects, promoting mitosis. However, elevated ROS levels can cause significant cellular damage [1]. To assess the magnitude of oxidative stress in hepatocytes, we quantified CAT, SOD, and ROS levels. Our study revealed a notable elevation in CAT and SOD activity, accompanied by a dose-dependent increase in ROS in the hepatocytes of the treated groups compared to the control. These elevations in CAT, SOD, and ROS are indicative of activated oxidative stress, suggesting that Cr(VI) induces oxidative stress in turtle hepatocytes.

Furthermore, Liang et al. [35] found that Cr(VI) stimulates the production of reactive oxygen species (ROS) in L-02 hepatocytes, disrupting mitochondrial dynamics and ultimately initiating hepatocyte apoptosis [31]. Mitochondria occupy a central role in cellular metabolic processes [36], serving as indispensable organelles within the cell. They generate stable energy for cellular functioning [37] and are important sites for ATP synthesis. Notably, mitochondria are not only the primary location for ROS production, but are also a target of ROS attack [38]. When the body fails to eliminate damaged mitochondria, excess ROS accumulates in cells. This accumulation leads to inhibited ATP production and disrupts mitochondrial homeostasis [39]. Research has indicated that ROS production is a pathway contributing to mitochondrial dysfunction [40]. In addition, SIRT1 plays a variety of key roles in cells, including the regulation of gene expression, cellular senescence, stress responses, and metabolism, and affects the activity of transcription factors through deacetylation, thus playing an important role in various physiological and pathological processes in cells [41]. SIRT3 is mainly localized in mitochondria and plays a role in the regulation of mitochondrial homeostasis and metabolism, and is also closely associated with mitochondrial autophagy, inflammatory response, and apoptosis [42,43]. Cr(VI) disrupts mitochondrial dynamics by inhibiting the Sirt1/PGC-1a pathway, ultimately triggering apoptosis and autophagy in rat kidney cells [44]. Mfn2 is a GTPase located in the outer membrane of mitochondria that regulates the dynamic homeostasis of mitochondria, maintains mitochondrial morphology by balancing fusion and division, and influences mitochondrial metabolism, which affects apoptosis [45]. In our study, ATP production levels exhibited a dose-dependent decrease, with a significant reduction observed in the 50 μM group. In addition, the mRNA levels of mitochondrial dynamics-related genes, specifically Mfn2 and SIRT1, were notably downregulated. Additionally, at a concentration of 50 μM, the mRNA level of the SIRT3 gene decreased significantly. These findings suggest that Cr(VI) possesses the capability to induce mitochondrial dysfunction and disrupt dynamic equilibria in turtle hepatocyte.

When Indo-Pacific humpback dolphin (Sousa chinensis) skin fibroblasts of are exposed to Cr(VI), it modulates the expression of anti-apoptotic (Bcl2) and pro-apoptotic (Bax) proteins, thereby affecting cellular mitochondrial membrane potential and resulting in a depletion of ATP levels [30]. Additionally, Cr(VI) exposure has been observed to up-regulate p53 and caspase3 expressions, thereby suppressing cell viability and triggering cell apoptosis [30]. p53 can both up-regulate the expression level of Bax and down-regulate the expression of Bcl-2 [46]. Bcl-2 and Bax proteins play contrasting roles in apoptosis, with Bcl-2 inhibiting and Bax promoting this cellular death process [47]. Conversely, autophagy serves as a cellular self-protection mechanism, essential for maintaining cell homeostasis and cell survival [48,49]. Normal autophagy levels shield cells from external stimuli, but imbalances can cause organelle damage, such as mitochondrial dysfunction, potentially leading to diseases [50]. The p53 protein also regulates autophagy, often via the mTOR signaling axis [50]. Apoptosis and autophagy work together to maintain cellular homeostasis [49,51]. Previous studies show that Cr(VI) exposure activates p53, Caspase-3, and alters the expression of Bcl2 and Bax, leading to apoptosis [30]. Both Beclin1 and LC3 proteins are hallmark proteins of autophagy. LC3 exists in two forms: LC3-I and LC3-II. LC3-I is the unmodified form and is found mainly in the cytoplasm. When the cell senses an autophagic signal, LC3-I is phosphorylated to LC3-II and binds to autophagic vesicle-associated membranes, indicating a protein specific for the degree of autophagy [52]. Beclin1 directs the localization of associated proteins to the autophagosome membrane by regulating the formation of autophagy precursors and regulates the balance between autophagy and apoptosis in cells [53,54]. p62/SQSTM1 is an important selective autophagic bridging protein that is itself degraded by autophagy and participates in the autophagic degradation of ubiquitinated protein aggregates in lysosomes [55]. Exposure to Cr(VI) significantly altered the expression of autophagy-related genes: LC3-I, LC3-II, Beclin-1 mRNA levels increased, while mTOR mRNA decreased, and p62/SQSTM1 protein levels dropped [56]. Our findings revealed a concentration-dependent increase in p53 gene expression with Cr(VI) exposure. Apoptosis-related genes Bax and Caspase3 mRNA levels rose significantly, while Bcl2 mRNA decreased, leading to a concentration-dependent rise in apoptosis. Furthermore, mRNA levels of autophagy-related genes LC3-I, LC3-II, ULK1, and p62/SQSTM1 surged, whereas mTOR and Beclin-1 mRNA levels fell. These results suggest that Cr(VI) exposure activates the p53 signaling pathway, thereby leading to abnormal overactivation of apoptosis and autophagy mechanisms.

## 5. Conclusions

Our findings revealed that Cr(VI) triggers apoptosis and autophagy in hepatocytes by inducing oxidative stress, mitochondrial dysfunction, and activating the p53 signaling pathway. However, our investigations were limited to in vitro experiments on turtle hepatocytes. Future studies on farm turtles are being considered to explore additional mechanisms.

## Data Availability

The raw data supporting the conclusions of this article will be made available by the authors on request.

## Supplementary Materials

The following supporting information can be downloaded at: https://www.mdpi.com/article/10.3390/ani14162403/s1, Supplementary Material Table S1: The primer sequences used in this study.
