# Supplementary material for: Chromium Affects Mitochondrial Function, Leading to Apoptosis and Autophagy in Turtle Primary Hepatocytes
Source: Animals (Basel). 2024 Aug 19;14(16):2403. doi: 10.3390/ani14162403 (PMC11350686; doi:10.3390/ani14162403)
Supplement: Supplementary file 1 [file animals-14-02403-s001.zip › animals-3130273-supplementary.pdf]

Table

Table S1. The primer sequences used in the this study.

Gene	Primer sequence (5'-3')
Mfn2	F: TGGCATTAGTGAGGTGCTGG<br>R: GAGAGCATGAGCCAGTTGGT
SIRT1	F: TTCCATCCCCCATGAAGTGC<br>R: GCCTTACGTTCAGTTGGATGC
SIRT3	F: GATGCAGTCCGCAGCTCTAT<br>R: TCCTGCATCTCCTCCTTCCA
LC3-I	F: GATCCGAGAGCAGCATCCAA<br>R: GGCGCCGGATAATTTTCACC
LC3-II	F: TGCAGCTGAACTCCACTCAG<br>R: TGATATGGGGGTTGACACGC
mTOR	F: ATGCGATCCAGCTGTTTGGA<br>R: GGACGTCTGGGGCATCAAA
ULK1	F: GCTCATTTTCAGCTACGCCG<br>R: CGCACAGATCCCCGATAACA
p62/SQSTM1	F:AGTCAGCAACTTCCTGTCAC<br>R: CTGGTCTAGGGGGAACCTGA
Beclin1	F: GGCAGAAGCAGAGAGACTGG<br>R: AACTGGATCTGGGCATAGCG
P53	F:CATGTGGATGCAAGGTGCTC<br>R:TGTGGTGGAATCCTTTCGGG
BAX	F: CGGTCTCGAAGGAGGTGTTT<br>R: TCCTGAGCGATGACCCTGTA
BCL-2	F: TGCTAGTAACGTGCCCCTTG<br>R: TCATCTCCAGCTTGGCACAG
Caspase-3	F: TGGACTGCAATCAGGTCACG<br>R: ACAGGCAGGCTCTTTCCTTG
β-actin	F: CCGTGATCTGACGGACTACC<br>R: TGTCACGCACGATTTCCCTT
